# Exploring age-related differences in the relationship between spatial and temporal contributions to step length asymmetry during split-belt adaptation

**DOI:** 10.1007/s00221-024-06929-1

**Published:** 2024-10-24

**Authors:** Patrick G. Monaghan, William M. Murrah, Kristina A. Neely, Harrison C. Walker, Jaimie A. Roper

**Affiliations:** 1 School of Kinesiology, Auburn University, Auburn, AL USA; 2 Department of Educational Foundations, Leadership, and Technology, Auburn University, Auburn, AL USA; 3 Department of Neurology, University of Alabama at Birmingham, Birmingham, AL USA

**Keywords:** Aging, Biomechanics, Walking, Coordination, Gait

## Abstract

Gait adaptability is crucial for meeting environmental demands, and impaired gait adaptation increases fall risk, particularly in older adults. While prior research exists on older adults’ gait adaptation, particularly in perturbation studies, the specific contributions of temporal and spatial adaptation strategies to step length asymmetry (SLA) during split-belt treadmill walking require further examination. This study fills this gap by evaluating how distinct adaptation strategies contribute to SLA in healthy young and older adults. 19 healthy young adults (20.4 ± 1.1 years) and 19 healthy older adults (68.3 ± 8.1 years) walked on a split-belt treadmill requiring their non-dominant leg to move twice as fast as their dominant leg. Repeated measures ANOVA investigated (1) spatial and temporal contributions to SLA, (2) SLA across gait adaptation epochs, and (3) rates of adaptation and deadaptation. Older adults displayed reduced temporal contributions to SLA compared to younger adults (F_1,36_ = 6.42, p = .02, ŋ^2^ = .15), but no group differences were observed in spatial contributions to SLA (F_1,36_ = 3.23, p = .08, ŋ^2^ = .082). SLA during adaptation and deadaptation did not differ by age group, nor did the rate of adaptation (F_1,34.7_ = 0.594, p = .45) or deadaptation F_1,33.6_ = 2.886, p = .09). These findings suggest that while older adults rely less on temporal strategies for gait adaptation, but maintain overall adaptability comparable to younger adults. Findings enhance our understanding of age-related changes in gait adaptation mechanisms and may inform targeted interventions to improve gait adaptability in older populations.

**Supplementary Information:**

The online version contains supplementary material available at 10.1007/s00221-024-06929-1.

## Introduction

Gait adaptations are crucial for meeting the demands of an ever-changing environment. Understanding how people adapt their steps during continuous walking when faced with internal or external perturbations is critical to permitting successful interactions within our surroundings (Choi and Bastian 2007a). Inadequate adaptations to internal perturbations or external environment cues compromise flexibility in navigating environmental challenges and can lead to an increased risk of falls in populations with motor impairments, which are a significant concern due to their association with injury, reduced independence, and compromised quality of life. Internal perturbations refer to challenges that arise from within the body, such as motor control adjustments and sensorimotor integration. External perturbations refer to challenges from outside the body, such as the differing speeds of the treadmill belts (Choi and Bastian 2007b; Darter et al. 2018; Fettrow et al. 2021). Common external perturbations in daily life include negotiating different surface types or obstacles within our path, while internal perturbations can include injury or different loading patterns (such as wearing a backpack). Spatial (where we place our foot) and temporal (when we place our foot) strategies can overcome such perturbations. Flexible adaptation of spatial and temporal gait patterns permits successful navigation of challenging and complex environments in daily life (Choi and Bastian 2007a).

Split-belt treadmill walking paradigms, in which individuals walk with their legs moving at different velocities, have often been used to examine and measure gait adaptation (Prokop et al. 1995; Reisman et al. 2005; Choi and Bastian 2007b; Vasudevan et al. 2017). The adaptation process we refer to here is primarily feedforward, involving the trial-and-error modification of an established movement pattern in response to novel task demands (Martin et al. 1996). Step length asymmetry (SLA) is a standard outcome metric reported as an index of gait adaptation performance (Reisman et al. 2005; Bruijn et al. 2012a; Malone et al. 2012). Initially, SLA increases in response to the differing belt speeds but gradually decreases after exposure to the continuous perturbation. When the belts are returned to the same speed, individuals depict negative aftereffects (SLA in the opposite direction)(Reisman et al. 2005). The absence of aftereffects after a period of split-belt walking is considered a sign of a reduced ability to adapt the gait pattern and may reflect declines in sensorimotor flexibility.

While SLA has traditionally been used as a marker of gait adaptation performance, it is important to recognize that symmetry may not be the ultimate goal during split-belt treadmill adaptation. Recent evidence suggests that the adaptation process involves a shift from negative asymmetry, where individuals take longer steps on the slow belt, to positive asymmetry, characterized by longer steps on the fast belt. This shift is believed to exploit the mechanical work provided by the treadmill, which may reduce the metabolic cost of walking (Sánchez et al. 2019). Furthermore, the observation of symmetry in previous studies may reflect a transient point within this shift, rather than an end goal of the adaptation process. This notion aligns with arguments that the length of gait trials can influence the degree to which symmetry is observed, as it represents a momentary balance before further adaptation occurs. Thus, understanding the dynamic nature of SLA during split-belt treadmill walking is crucial, as it highlights the importance of both spatial and temporal adaptations beyond the pursuit of symmetry alone.

While SLA is a classic marker of gait adaptation ability, recent work has recognized SLA as a hybrid measure comprised of spatial and temporal components (Finley et al. 2015a; Roper et al. 2019a; Brinkerhoff et al. 2021a; Gregory et al. 2021). Specifically, the adaptation of SLA involves alterations in the spatial control of foot placement and the temporal control of stepping timing (Finley et al. 2015a; Long et al. 2016a). These spatial and temporal adaptation mechanisms are evident in everyday life. For example, one may adjust foot placement when avoiding a puddle or stepping up a curb, while step timing may be adjusted when we are required to avoid other people or obstacles in our line of progression. The spatial and temporal contributions to SLA reflect distinct neural strategies individuals engage to overcome the asymmetric belt speeds during split-belt treadmill walking (Malone and Bastian 2010a; Malone et al. 2012). Recent studies have also documented that these spatial and temporal strategies may be controlled independently (Gonzalez-Rubio et al. 2019a; Darmohray et al. 2019a). Therefore, a greater understanding of the spatial and temporal contributions to SLA will provide greater insight into specific strategies and neural processes employed to adapt a novel walking pattern.

Age-related declines in physiological and cognitive functions can significantly impair gait adaptation in older adults (Caetano et al. 2017). For instance, reductions in muscle strength and power can limit the force generation necessary for effective gait adjustments (Stotz et al. 2023), particularly during challenging conditions like split-belt treadmill walking. Additionally, older adults often experience visuomotor deficits (Gomez-Granados et al. 2021; Li et al. 2021), which impair the processing and integration of visual information crucial for adapting to new walking environments. Furthermore, decreased confidence in walking (MacKay et al. 2021), frequently stemming from a history of falls or fear of falling, can result in more conservative and less effective adaptation strategies. The cerebellum plays a critical role in adapting gait patterns during split-belt walking, as evidenced by studies showing a positive correlation between cerebellar excitability and increased step length symmetry (Jayaram et al. 2011). Additionally, enhancing cerebellar function through transcranial direct current stimulation has been shown to improve gait symmetry during split-belt walking (Jayaram et al. 2012). These findings suggest that impaired adaptability in gait, particularly in older adults, may be linked to age-related declines in the structural integrity of cortico-cerebellar pathways, which are crucial for motor learning and adaptation (Hogan 2004). These factors may collectively contribute to slower adaptation rates and diminished aftereffects in older adults compared to their younger counterparts.

While recent work in clinical populations has examined the spatial and temporal contributions to SLA during split-belt treadmill walking (Finley et al. 2015a; Roper et al. 2019a), much less is known concerning age-related differences in spatial and temporal gait adaptation strategies. Older adults can adapt their gait to a novel continuous perturbation (Bruijn et al. 2012b; Roemmich et al. 2014a; Malone and Bastian 2016; Sombric et al. 2017; Ducharme et al. 2019; Vervoort et al. 2019). However, they do so more slowly and are less able to temporarily retain the adjustments (Bruijn et al. 2012b; Sombric et al. 2017). Sato and colleagues reported that timing adaptation differed between older and younger adults, but step length asymmetry did not (Sato and Choi 2022). However, the relative contributions of spatial and temporal strategies across different stages of adaptation remain unclear. Specifically, there is a gap in our understanding of how the balance between spatial and temporal control strategies may shift during early, mid, and late stages of adaptation in older adults compared to younger individuals. This knowledge is crucial as it may reveal age-specific compensatory mechanisms or limitations in gait adaptability that are not apparent when examining overall performance metrics alone. This novel approach allows us to examine whether older adults rely more heavily on one strategy over another at different points in the adaptation process, potentially revealing age-specific patterns of motor adaptation.

Here we characterize age-related differences in the relative contributions of spatial and temporal control strategies during continuous gait adaptation. The overarching goal is to examine whether spatial and temporal strategies differ between healthy young and older adults during three distinct adaptation phases in split-belt treadmill walking (i.e., early-, mid-, and late-stage). We also compared gait adaptation at baseline and during both adaptation and de-adaptation in healthy older and younger adults. We hypothesize that older adults display altered spatial and temporal contributions to SLA during early, mid, and late adaptation based on work highlighting potential age-related differences in gait adaptation strategies (Bruijn et al. 2012b; Vervoort et al. 2019; Stenum and Choi 2020). Further, based on prior research (Bruijn et al. 2012b; Malone and Bastian 2016; Vervoort et al. 2019), we hypothesize that there will be no age-related differences in SLA between older and younger adults adapt SLA. However, we expect that older adults will demonstrate slower adaptation rates and exhibit less pronounced aftereffects during de-adaptation compared to younger adults.

## Materials and methods

### Participants

We recruited 19 healthy young adults and 19 healthy older adults aged 55 or older (Table 1). All participants completed written informed consent prior to study participation. Sample size of 19 per group was determined based upon effect sizes from previous literature (Bruijn et al. 2012b) using F-test power analysis within G*Power 3.1, assuming β error probability of 0.8 and α error probability of 0.05. Participants were free from cardiovascular, pulmonary, renal, metabolic, vestibular, and neurologic disorders and reported no lower-extremity injuries or surgeries in the past 12 months that might limit their capacity to complete the protocol or alter their gait. All participants reported being able to walk unassisted without balance issues, and all were naïve to split-belt treadmill walking. The protocol was approved by the Auburn University Institutional Review Board before any subject enrollment and performed in accordance with the ethical standards laid down in the 1964 Declaration of Helsinki.

**Table 1:** Participant characteristics

	Overall (n = 38)	Young adults (n = 19)	Old adults (n = 19)	P-value
Age (years)	44 (25)	20 (1)	68 (8)	< .001
Sex	30 F, 8 M	14F, 5 M	16 F, 3 M	.27
Height (m)	1.69 (0.1)	1.72 (0.09)	1.65 (0.1)	0.02
Mass (kg)	66.82 (12.07)	66.86 (11.66)	68.26 (8.11)	0.98
Typical walk speed (m/s)	1.13 (0.24)	1.26 (0.20)	0.99 (0.21)	< .001
Normalized typical walk speed^†^	0.67 (0.14)	0.73 (0.12)	0.60 (0.13)	0.008
Fast walk speed (m/s)	1.42 (0.25)	1.58 (0.15)	1.26 (0.23)	< .001
Normalized fast walk speed^†^	0.85 (0.15)	0.93 (0.11)	0.77 (0.14)	< .001
MMSE	28.86 (1.08)	28.95 (1.03)	28.78 (1.16)	0.64
Digit span- forward	10.05 (2.39)	9.84 (2.34)	10.26 (2.49)	0.6
Digit span- backward	7.05 (2.12)	6.47 (1.98)	7.63 (2.14)	0.09
TMT- A (s)	23.91 (10.66)	17.53 (4.24)	30.29 (11.38)	< .001
TMT-B (s)	52.84 (26.86)	39.77 (12.72)	66.63 (31.04)	0.002

Values are reported as mean (SD)

*MMSE* mini-mental state exam, *TMT* trails making test

†Typical and fast walking speed divided by height due to differences in height bewteen groups

### Experimental protocol

Before treadmill testing, all participants completed cognitive tests including the Mini-Mental State Exam, Digit Span Forward and Backward, and Trails Making Test A and B (Blackburn and Benton 1957; Folstein et al. 1983; Bowie and Harvey 2006). All cognitive tests were performed in the same order and by the same administrator. Retroreflective markers were then placed according to the Vicon Plug-In Gait Full Body Ai Functional model, and kinematic data were collected at 100 Hz (VICON; Vicon Motion Systems Ltd, Oxford, United Kingdom). Next, participants began testing on the split-belt treadmill. Participants were instructed to hold on to the handrails for all trials. We first obtained each participant’s typical and fast walking speeds while they walked on the treadmill with both belts moving at the same speed. To calculate comfortable walking speed, the belts began at a relatively slow speed and then increased until the participants reported reaching their typical speed. This was repeated twice, and the average of the reported values was recorded as the participant’s typical walking speed. Similarly, to calculate the participant’s fast walking speed, the belts began at a relatively slow speed and increased in speed until the participants felt like they were walking at the fastest speed that they could comfortably walk at for ten minutes. Two trials were recorded, and the average value was reported at the participant’s fastest walking speed. The participant’s fastest walking speed was set as the fast belt speed, and half of the fastest comfortable speed was set as the slow belt speed. Previous studies have undertaken a similar methodology to determine fast and slow belt speeds (Dingwell and Marin 2006; Roemmich et al. 2014b). To determine leg dominance, we asked participants which leg they would use to kick a soccer ball. We placed their self-reported dominant leg on the slow belt of the treadmill.

Participants then walked for three minutes with both belts at their typical walking speed, followed by three minutes with both belts at their fastest and three minutes at slow walking speed (BASELINE). This three-minute walking trial at slow walking speed served as the baseline trial, as split-belt literature has recommended matching the speed of baseline trials to the speed at which the after-effects will be tested during the deadaptation phase. It has been shown that the largest treadmill after-effects occur when the tied-belt speed matches that of the slower belt during split-belt adaptation (Vasudevan and Bastian 2010; Vasudevan et al. 2017). Next, the belt under the participant’s non-dominant leg was increased to their fastest walking speed while the belt under the dominant leg remained at their slow walking speed. Participants walked under these conditions for ten minutes (ADAPT) and subsequently walked with both belts at the slow speed for three more minutes (DEADAPT). The belts were stopped temporarily between each treadmill condition before initiating the next condition.

### Data analysis

All treadmill walking trials took place on a Bertec instrumented spit-belt treadmill (Bertec, Columbus, OH, USA). Kinematic data were filtered using a low-pass fourth-order Butterworth filter with a cutoff frequency of 6 Hz. Step length was calculated as the anteroposterior distance between the ankle markers at foot strike. SLA was calculated and normalized to stride length using the following equation:

$$Step length Asymmetry= \frac{{Step length}_{fast}-{Step length}_{slow} }{{Step length}_{fast}+{Step length}_{slow}}$$

Step Length_fast_ refers to when the leg in the fast belt strikes the belt, and Step Length_slow_ refers to when the leg on the slow belt strikes the belt. Positive asymmetry indicates that the leg on the fast belt is taking a longer step, and negative asymmetry indicates that the leg on the slow belt is taking a longer step. An asymmetry value of zero indicates that the legs are taking steps of equal length. The calculation of SLA is the same equation as used in previous studies that have examined SLA during split-belt treadmill walking (Bruijn et al. 2012b; Roemmich et al. 2014b; Malone and Bastian 2016; Roper et al. 2019b). Previous studies have demonstrated that SLA can be decomposed into a sum of contributions related to space, timing, and velocity (Finley et al. 2015b; Long et al. 2016b). These measures encompass the location, timing, and velocity of the feet during walking. Accordingly, we can calculate SLA’s spatial, temporal, and velocity contributions.

$$Step length Asymmetry=\left({\propto }_{f}- {\propto }_{s}\right) +\left[\frac{{v}_{s}+{v}_{f}}{2} X \left({t}_{s}-{t}_{f}\right)\right] + \frac{{t}_{s}+{t}_{f}}{2} X ({v}_{s}-{v}_{f})$$

The first term represents the spatial contribution to SLA based on where participants placed their feet relative to their bodies at heel strike. ${\propto }_{f}$ refers to where the fast foot is placed relative to the previous slow foot placement, while ${\propto }_{s}$ refers to where the slow foot is placed relative to the previous fast foot placement. The second term represents timing contributions to SLA based on step timing differences. ${t}_{s}$ refers to the time between a slow foot heel strike and the previous fast foot heel strike while ${t}_{f}$ is the time between a fast foot heel strike and the previous slow foot heel strike. Additionally, ${v}_{s}$ is the average velocity of the slow ankle relative to the body during the slow step time, while ${v}_{f}$ is the average velocity of the fast ankle relative to the body during the fast step time. The final term represents velocity contributions related to the differences in belt speeds. The spatial, temporal, and velocity contributions to SLA were derived from non-normalized SLA values.

SLA values and the contributions to SLA were calculated at five different epochs throughout the split-belt treadmill paradigm: (1) the average of the last five strides of baseline at slow walking speed (BASELINE), (2) the average of the first five strides of ADAPT (Early-ADAPT), (3) the average of the middle five strides of ADAPT (Mid-ADAPT), (4) the average of the last five strides of ADAPT (Late-ADAPT) and (5) the average of the first five strides of DEADAPT.

Rates of adaptation and deadaptation were calculated as the number of steps a participant took until five consecutive strides were within two standard deviations of the plateau during adaptation and deadaptation. Plateaus were defined as the mean of the last thirty strides of adaptation and deadaptation, as described previously (Finley et al. 2015b; Brinkerhoff et al. 2021b).

### Statistical analyses

All statistical tests were conducted using SPSS Statistics version 26 (IBM, Armonk, New York). One-way ANOVA analyzed demographic variables (Table 1), and repeated measures ANOVA contrasted differences in spatial and temporal contributions to SLA by age group (old versus young adults) and across adaptation epochs (Early-ADAPT, Mid-ADAPT, Late-ADAPT). Second, repeated measures ANOVA contrasted differences in SLA by age group (young versus old adults) and adaptation epochs (BASELINE, Early-ADAPT, Mid-ADAPT, Late-ADAPT, and DEADAPT).

To examine adaptation and deadaptation rates, we used repeated measures ANOVA across age groups (old versus young adults) and epoch (adaptation versus deadaptation). All analyses accounted for differences in the fastest walking speed and baseline asymmetries. Analyses of subtracting each participant’s initial baseline asymmetry from their subsequent gait measurement did not alter the study outcomes, ensuring that any observed effects were specifically due to the experimental conditions. For each statistical procedure, the significance level was α = 0.05, and Tukey post-hoc adjustments were applied when appropriate. Partial eta-squared (ŋ^2^) values are also reported as an index of effect size.

Before conducting the analyses, we verified normality and visually inspecting histograms, assessed for skewness and kurtosis, and conducted Shapiro–Wilk tests. We also investigated extreme data points that exceeded two standard deviations from the mean. However, excluding these data points did not alter the statistical inference or interpretation of the results. To assess the assumption of sphericity, we utilized Mauchly’s test. If Mauchly’s test indicated a violation of the sphericity assumption, we relied on adjusted degrees of freedom based on the Greenhouse–Geisser correction.

## Results

### Effect of age on temporal and spatial contributions to SLA during gait adaptation

**Fig. 1:**
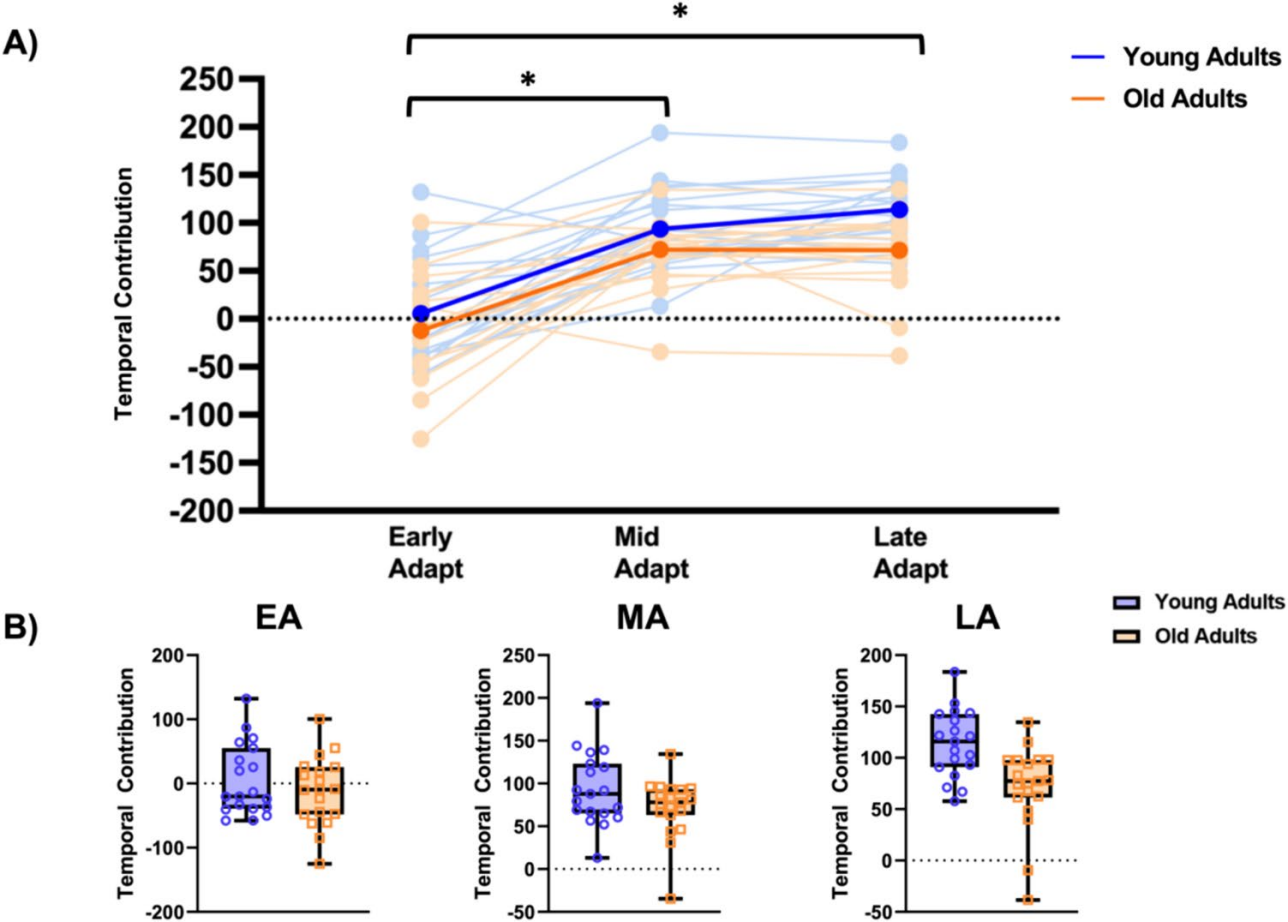
Age-related differences in temporal contributions to SLA during gait adaptation epochs. **A** Light blue lines depict younger adults, while light orange lines represent older adults. The darker filled blue lines and dark filled orange lines represent the mean values for younger and older adults, respectively. **B** Box plots illustrate temporal contribution to SLA across gait adaptation epochs. Individual participants are indicated by open filled circles and squares. *EA* early adaptation, *MA* middle adaptation, *LA* late adaptation. *p < .001

Repeated measures ANOVA compared temporal contributions to SLA between age group adaptation epoch, revealing a main effect of epoch (F_1.46,52.63_ = 83.22, p < 0.001, ŋ^2^ = 0.70). Post-hoc pairwise comparisons with Tukey correction indicated that irrespective of group, temporal contributions to SLA increased during Mid-Adapt (Mdiff = −86.43 mm, SE = 9.52, p < 0.001) and Late-ADAPT (Mdiff = −96.20, SE = 9.21, p < 0.001). Temporal contributions did not significantly differ between Mid-ADAPT and Late-ADAPT (Mdiff = −9.77, SE = 5.16, p = 0.155). We also observed a significant main effect of age group (F_1,36_ = 6.42, p = 0.02, ŋ^2^ = 0.15). Post-hoc pairwise comparisons with Tukey correction indicated that irrespective of epoch type, older adults use less temporal contributions to SLA than younger adults (Mdiff = 27.1, SE = 10.7, p = 0.02). No significant age group x epoch interaction was observed (F_1.46,52.63_ = 1.33, p = 0.27, ŋ^2^ = 0.036) (Fig. 1).

**Fig. 2:**
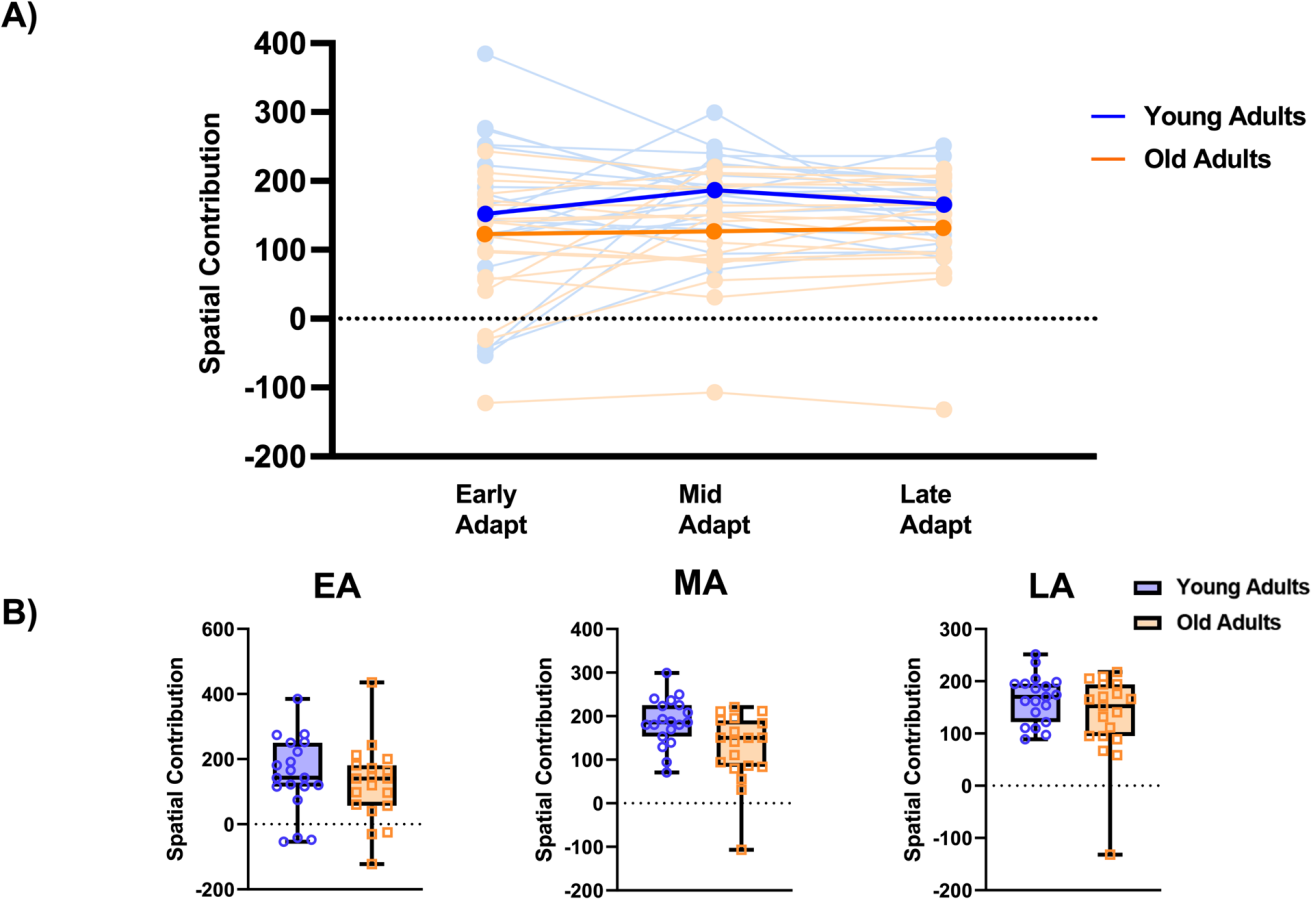
Age-related differences in spatial contributions to SLA during gait adaptation epochs. **A** Spatial contribution changes during gait adaptation epochs. Light blue lines depict young adults, and light orange lines represent older adults. The darker filled blue and orange lines represent the group means for younger and older adults, respectively. **B** Box plots illustrate spatial contributions to SLA across gait adaptation epochs. Individual younger adults are represented by purple open filled circles, and individual older adults are represented by open filled orange squares. *EA* early adaptation, *MA* middle adaptation, *LA* late adaptation

Repeated measures ANOVA showed no significant main effect of adaptation phases or epoch on spatial contribution to SLA (F_1.22,43.90_ = 0.893, p = 0.37, ŋ^2^ = 0.024), or age group (F_1,36_ = 3.23, p = 0.08, ŋ^2^ = 0.082), nor did it show group x epoch interactions (F_1.22,43.90_ = 0.664, p = 0.45, ŋ^2^ = 0.018). (Fig. 2).

### Effect of age on SLA during baseline, early, mid, late adaptation, and deadaptation

**Fig. 3:**
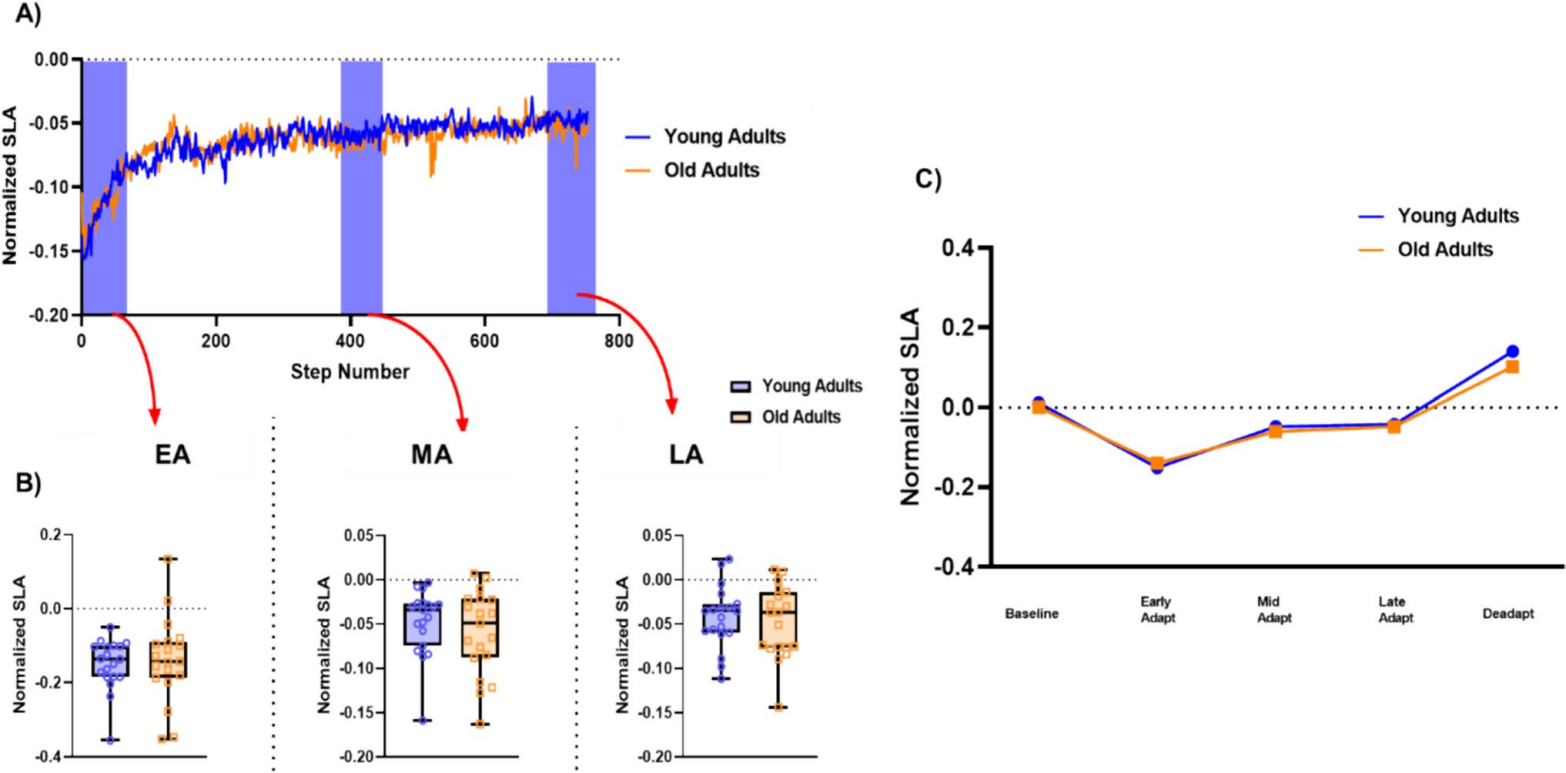
Age-related variations in SLA across epochs of walking adaptation. **A** Step-by-step SLA values, averaged for 19 older and 19 younger adults during novel gait adaptation. The orange lines depict older adults, while the blue lines represent younger adults. Shaded areas indicate distinct epochs of gait adaptation (early, mid, and late adaptation). **B** Box plots illustrating SLA values during early, mid, and late gait adaptation. Individual younger adults are denoted by purple open filled circles, and individual older adults are denoted by open filled orange squares. **C** Average SLA values across different epochs, presented by the blue line for younger adults and the orange line for older adults. *EA* early adaptation, *MA* middle adaptation, *LA* late adaptation

Repeated measures ANOVA compared SLA by age group and adaptation epoch, revealing a significant main effect of epoch (F_1.69, 64.34_ = 129.634, p < 0.001, ŋ^2^ = 0.77). Post-hoc pairwise comparisons with Tukey correction indicated that irrespective of age group, compared to BASELINE, SLA differed by epoch, except between Mid- and Late-ADAPT (see summary results in Table 2). No significant main effect of Group was observed (F_1,38_ = 0.583, p = 0.45, ŋ^2^ = 0.015), nor was there a significant group x epoch interaction (_F1.69, 64.34_ = 0.719, p = 0.47, ŋ^2^ = 0.02) (Fig 3).

**Table 2:** Main effect of epoch. Tukey-adjusted post hoc comparisons

Comparison			Mean difference	SE	df	t	p_tukey_
Epoch		Epoch					
Baseline	-	Early adapt	0.150	0.016	38.0	9.62	** < .001**
	-	Mid adapt	0.061	0.007	38.0	9.37	** < .001**
	-	Late adapt	0.052	0.006	38.0	9.17	** < .001**
	-	Deadapt	−0.112	0.011	38.0	−10.40	** < .001**
Early adapt	-	Mid adapt	−0.089	0.012	38.0	−7.52	** < .001**
	-	Late adapt	−0.098	0.013	38.0	−7.49	** < .001**
	-	Deadapt	−0.261	0.020	38.0	−13.02	** < .001**
Mid adapt	-	Late adapt	−0.009	0.004	38.0	−2.39	0.139
	-	Deadapt	−0.173	0.012	38.0	−14.58	** < .001**
Late adapt	-	Deadapt	−0.163	0.011	38.0	−15.11	** < .001**

Significant differences highlighted in **bold**

### Rate of adaptation and deadaptation

**Fig. 4:**
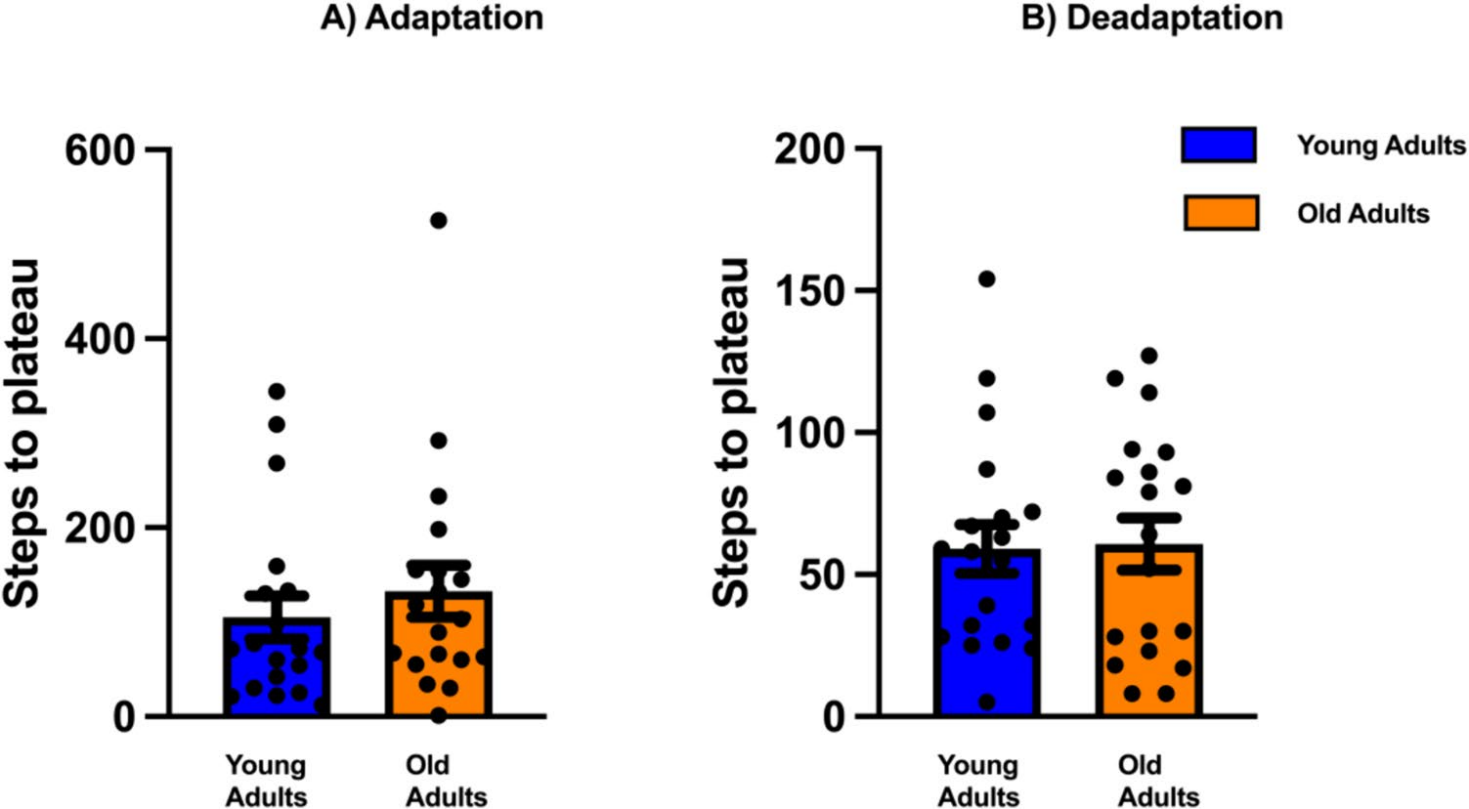
Age-related differences in the rate of adaptation and deadaptation. The average number of steps to plateau during **A** adaptation and **B** deadaptation for older and younger adults. Blue bar depicts younger adults and orange bar represents older adults. Solid filled circles represent individual data points

One-way ANOVA compared the rate of adaptation and deadaptation between older and younger adults. Our results revealed no significant differences in either the rates of adaptation (F_1,34.7_ = 0.594, p = 0.45) or deadaptation (F_1,33.6_ = 2.886, p = 0.09) (Fig. 4).

## Discussion

This study investigated how age affects spatial and temporal contributions to gait adaptation during novel walking tasks on a split-belt treadmill. Our study is novel as we compare the spatial and temporal strategies during distinct time points of the adaptation paradigm. We contrasted age-related changes in gait adaptation performance and the rate of adaptation and deadaptation in otherwise healthy adults. Our main findings are: (1) Older adults rely less on temporal contributions for gait adaptation on the split-belt treadmill than younger adults, while spatial contributions are similar between the two groups. (2) Older adults adapt and retain temporal adjustments to walking patterns and do so at the same rate as younger adults. These findings support our hypothesis that older and younger adults utilize different spatial and temporal strategies during novel gait adaptation. Our second hypothesis, which assumed no age-related differences in adaptation, is partially supported as there was no difference in SLA values across the epochs of adaptation between older and younger adults. However, our initial expectation of reduced rates of adaptation, deadaptation, and aftereffects in older adults was not observed.

 Older adults rely less on temporal contributions to gait adaptation than younger adults, while no age-related differences were observed in spatial contributions. This suggests that older and younger adults employ different strategies to achieve adaptation of SLA, with older adults tending to adjust their step position instead of step timing. Previous research has demonstrated that spatial and temporal interlimb measures adapt at different rates and can be altered independently (Malone and Bastian 2010b; Long et al. 2016c; Sombric et al. 2017; Gonzalez-Rubio et al. 2019b; Darmohray et al. 2019b; Sato and Choi 2022). Therefore, distinct neural circuits might underpin temporal and spatial strategies, hence permitting the finite adjustments made to limb movements (Gonzalez-Rubio et al. 2019c; Darmohray et al. 2019b). Cerebral mechanisms have been implicated in spatial and temporal coordination and may indirectly impact temporal coordination by altering brainstem or cerebellar circuits (Morton and Bastian 2006; Malone and Bastian 2010b, 2016). Documented age-related degradation in the integrity of the cortico-cerebellar pathways may explain the temporal decrements observed in older adults (Good et al. 2001; Smith et al. 2007). Our findings align with studies in clinical populations with possible cerebellar dysfunction, including patients with essential tremor. These individuals show reduced temporal contributions to gait adaptation although similar overall adaptation (Roper et al. 2019b). This supports the hypothesis of age-related alterations in cerebellar circuitry contributing to temporal dysregulation. This study provides a novel characterization of age-related differences in the underlying motor control mechanisms driving gait adaptability. Additionally, linking these age-related differences in spatial versus temporal adaptation strategies to potential neural mechanisms, such as declines in cortico-cerebellar pathways, represents a novel integration of behavioral and neurophysiological perspectives on gait adaptation across the lifespan.

The current study provides novel insights into how older and younger adults utilize distinct spatial and temporal strategies to adapt their gait during the different phases of split-belt treadmill walking. Importantly, we observed that while the overall magnitude of SLA did not differ between age groups, the underlying spatial and temporal contributions to SLA adaptation varied significantly. Specifically, we found that older adults relied less on temporal adjustments compared to younger adults, particularly during the mid and late stages of adaptation. This suggests that older individuals may adopt a more, spatially oriented strategy to overcome the novel walking demands, focusing more on modulating foot placement rather than step timing. In contrast, younger adults exhibited greater temporal flexibility, allowing them to dynamically adjust the relative timing of their steps to a larger degree. These age-related differences in the temporal versus spatial emphasis of gait adaptation strategies likely reflect distinct neural mechanisms that govern these control processes. Examining the temporal and spatial contributions to SLA across the different stages of adaptation therefore, provides a more nuanced understanding of the compensatory mechanisms older adults employ when faced with challenging gait perturbations, with important implications for targeted rehabilitation approaches.

Our findings indicate that age does not affect the capacity to adapt or temporarily retain adjustments to SLA during split-belt treadmill walking. This corroborates previous studies reporting no age-related differences in the magnitude of adaptation (Roemmich et al. 2014a; Malone and Bastian 2016; Ducharme et al. 2019; Iturralde and Torres-Oviedo 2019; Vervoort et al. 2019) but contrasts previous work that reported reduced aftereffects in SLA among older adults (Bruijn et al. 2012b). The outcomes of our study align with those of Iturralde and colleagues, who demonstrated that the magnitude of aftereffects is not correlated with age (Iturralde and Torres-Oviedo 2019). Moreover, we found no differences in the rate at which SLA adapts or washes out between healthy older and younger adults. Our results highlight that SLA was not a sensitive measure of distinguishing gait adaptation between older and younger adults. However, consideration of spatial and, in particular, temporal strategies was able to provide such a distinction. Therefore, future studies should seek to examine both spatial and temporal outcomes when examining age-related alterations in gait adaptation.

This study has several potential limitations. Although gait speed was not a significant covariate in our model, future studies should control for walking speeds to minimize the influence of belt-speed differences on adapting spatial and temporal aspects of gait during split-belt treadmill walking. The use of personalized walking speeds rather than a fixed 2:1 ratio may impact the interpretation of the results, as it introduces variability in speed ratios across participants. However, this approach was chosen to better align the task demands with each participant’s functional capacity and to minimize potential confounding factors such as fatigue or discomfort. By personalizing the speeds, we aimed to maintain a consistent level of challenge relative to each participant’s capabilities. Further information on fast and slow belt speeds for each participant can be found in supplemental material 1. Moreover, our participants held onto the handrail during all treadmill walking conditions, which might have impacted their stability and, consequently, their patterns of gait adaptation. However, this ensured better safety throughout the experimental protocol. Further, while this may limit the generalizability of our findings to some extent, it ensured internal validity by ensuring that all participants received the same instructions. It is also important to consider the restricted time allocated for adaptation, which may have prevented us from observing the full extent of participants’ gait adaptation. With additional time, it is possible that the adaptation could have continued to progress, potentially resulting in greater positive asymmetry, and the observed plateaus may not represent the true extent of adaptation. Furthermore, our findings, derived from treadmill-based walking, may not fully transfer to overground walking. As previous research suggests, including the work by Sanchez et al., treadmill walking may involve different biomechanical strategies that could affect the generalizability of our results to real-world walking scenarios.

## Conclusions

Older adults can adjust their walking patterns and retain adaptations temporarily to a novel walking pattern like younger adults. However, older adults tend to rely less on temporal strategies than younger adults; both age groups can use spatial strategies. While previous studies investigate the impact of age on gait adaptation, our study offers new and valuable insights into the spatial and temporal strategies that drive and enable gait adaptation on a novel split-belt treadmill. The insights gained from examining age-related differences in spatial and temporal gait adaptation strategies have important clinical implications. These findings can guide the development of more targeted rehabilitation programs for older adults, with a focus on enhancing temporal control of stepping rather than just spatial aspects. Additionally, understanding the age-related decline in temporal adaptation abilities may help explain reduced mobility and increased fall risk in older populations, suggesting this is an important therapeutic target.

## Supplementary information

Below is the link to the electronic supplementary material.

Supplementary file1 (XLSX 10 KB)

## Data Availability

Data sets generated during the current study are available from the corresponding author on reasonable request.
